# Caregiver burden and health-related quality of life in idiopathic dystonia patients under botulinum toxin treatment: a cross-sectional study

**DOI:** 10.1007/s00702-019-02109-6

**Published:** 2019-12-04

**Authors:** S. C. Drexel, M. Klietz, K. Kollewe, L. Paracka, A. Kutschenko, B. Kopp, F. Lange, F. Wegner, D. Dressler

**Affiliations:** 1 Department of Neurology, Hannover Medical School, Carl-Neuberg-Str. 1, 30625 Hannover, Germany; 2 Behavioral Engineering Research Group, KU Leuven, Naamsestraat 69, 3000 Leuven, Belgium

**Keywords:** Dystonia, Botulinum toxin, Quality of life, Caregiver burden, Depression, Anxiety

## Abstract

Dystonia is a chronic movement disorder that is associated with a reduction in health-related quality of life (HR-QoL) and restriction of activities of daily living. Botulinum neurotoxin (BT) improves disease-specific HR-QoL by reducing abnormal movements, postures, and pain. We examined the burden of the corresponding primary caregiver as a potential important factor for disease management and HR-QoL of dystonia patients under treatment with BT. 114 patients with focal, segmental, or generalized dystonia were recruited, together with 93 corresponding caregivers, whose burden was investigated using the Caregiver Burden Inventory. In addition, all participants were assessed for cognitive impairment, depression, anxiety, alexithymia, and HR-QoL. Only a small proportion of caregivers suffered from caregiver burden. Despite BT therapy, patients’ HR-QoL was decreased compared to the age-matched general German population. Psychological symptoms, notably anxiety, and depression correlated significantly with reduced HR-QoL. Our data imply that caregiver burden emerged to be an issue in subgroups of dystonia patients. Furthermore, HR-QoL of dystonia patients is reduced even under optimized BT treatment in a specialized center.

**Electronic supplementary material:**

The online version of this article (10.1007/s00702-019-02109-6) contains supplementary material, which is available to authorized users.

## Introduction

Dystonia is a chronic movement disorder characterized by abnormal twisted postures, sustained muscle hyperactivity, and pain (Dressler and Benecke 2005). Such dystonic movements are often visible and stigmatizing. Dystonia is classified based on somatic distribution of symptoms (focal, segmental, or generalized), age of disease onset (early or late), and etiology (primary or secondary) (Albanese et al. 2013; Geyer and Bressman 2006). Depending on the population assessed and the methods of ascertainment, focal dystonia affects approximately 8.4–11.9 per 100,000 people (Castelon Konkiewitz et al. 2002). Patients suffering from dystonia report problems with mobility, self-care, or usual daily activities (Gudex et al. 1998), and they also show distinct sensory (Paracka et al. 2017) and cognitive (Jahanshahi 2017; Lange et al. 2016a, b) alterations. Overall, dystonia is mainly seen as a disorder with primary motor symptoms; however, patients can also be affected by consecutive symptoms such as pain and a range of psychological symptoms such as mood disorders.

The treatment of dystonia is symptomatic including botulinum neurotoxin (BT) injections, oral medications, and deep brain stimulation (Dressler et al. 2016). BT is the first line therapy for most types of dystonia and can prevent complications like contractures (Benecke and Dressler 2007; Castelão et al. 2017; Marques et al. 2016). Cervical dystonia without BT treatment is associated with a reduced health-related quality of life (HR-QoL) as measured by the SF-36 (Mordin et al. 2014). The SF-36 scale measures the disease impact on the patient’s activities of daily living, physical, emotional, and social functioning, and is used in many studies assessing HR-QoL in dystonia (Gudex et al. 1998; Müller et al. 2002). It was repeatedly shown that SF-36 scores of untreated dystonia patients in the UK or USA are lower compared to healthy controls (Gudex et al. 1998; Mordin et al. 2014). Other studies showed that under treatment with BT, there is an improvement of disease-specific impairment (Castelão et al. 2017; Marques et al. 2016), HR-QoL (Kongsaengdao et al. 2017), pain, and depression (Weiss et al. 2017). These effects are strongest in the middle of the treatment cycle when BT is exerting its maximal effect. However, the BT effect on HR-QoL as measured by the SF-36 was also described as either mostly statistically insignificant (Gudex et al. 1998) or nonexistent (Kongsaengdao et al. 2017). Dystonia may relate to reduced HR-QoL not only in the affected patients, but also in their caregivers.

Recent studies have shown that caregiver burden is an important factor related to patients’ and caregivers’ HR-QoL in chronic movement disorders such as Parkinson’s disease (Klietz et al. 2019; Schmotz et al. 2017). The concept of caregiver burden is a multidimensional construct that summarizes the adverse effects of giving care for a person (e.g., financial, emotional, social, psychological, and physical) on the functioning of the caregiver (Zarit et al. 1986). Currently, little is known about burden of caregivers of dystonia patients in Germany. Therefore, we examined the HR-QoL of all subtypes of patients with idiopathic dystonia and their corresponding primary caregivers—as well as the related burden—as potentially important modulators of the management of dystonia in a cross-sectional design.

## Methods

### Participants

We obtained approval from the local Ethics Committee of Hannover Medical School (No. 7927_BO_K_2018), and all patients as well as their caregivers gave written informed consent. We enrolled 114 patients with dystonia and their caregivers at Hannover Medical School. 21 caregivers did not complete all questionnaires which renders a final sample of *N* = 114 patients and *N* = 93 caregivers. Inclusion criteria were defined as adult age of patient and caregiver, neurologically confirmed diagnosis of idiopathic dystonia and a non-professional caregiving situation at home. Patients with professional care or a neurological diagnosis of dementia were excluded from this analysis. The overwhelming majority of patients (98.3%) was under treatment with BT in 3-month intervals.

### Measures

All patients were seen by one trained examiner at the day that they received BT injections and dystonic symptoms were evaluated by Burke–Fahn–Marsden Dystonia Rating Scale (BFMDRS-I motor score, BFMDRS-II disability score) (Burke et al. 1985). Furthermore, the German version of Montreal Cognitive Assessment (MoCA) was used to evaluate cognitive functioning (Nasreddine et al. 2005). All patients suffering from a cervical dystonia were also assessed with the Toronto Western Spasmodic Torticollis Rating Scale (TWSTRS) (Consky et al. 1990) and the craniocervical dystonia questionnaire (CDQ-24) for disease-specific HR-QoL (Mueller et al. 2016). All participants (patients and their caregivers) were asked to complete the WHO Short Form 36 (SF-36) Health Survey for assessment of their HR-QoL (Ware and Donald-Sherbourne 1992), the Beck Depression Inventory (BDI) (Beck et al. 1961), the Toronto-Alexithymia Scale (TAS-26) (Bagby et al. 1994), and the STAI (STAI-S state anxiety and STAI-T trait anxiety) (Spielberger et al. 1983). In addition, primary caregivers were asked to complete the Caregiver Burden Inventory (CBI) (Zarit et al. 1980). The CBI comprises 22 items and a total score ranging from 0 (lowest burden) to 88 (highest burden), less than 20 points, suggest the absence of significant burden, 21–40 points are considered indicating mild, 41–60 points indicating moderate, and 61–88 points indicating severe burden. Patients’ and caregivers’ age, educational level, and relationship to caregiver were also recorded, together with patients’ years since diagnosis and the caregivers’ daily amount of time.

### Analyses

We performed a univariate regression model for the association of average SF-36 scores with sum scores of investigated predictors (Supplement Table 1). All items that were significant with *p* < 0.05 were included in a multivariate regression model to test against co-correlations and confounding. A back-to-back selection to the significance level of 5% was carried out. Furthermore, we performed a univariate regression model for the association of CBI scores with sum scores of investigated predictors (Supplement Table 2). All items that were significant with *p* < 0.05 were included in a multivariate regression model to test against co-correlations and confounding. A back-to-back selection to the significance level of 5% was carried out. To explore significant differences between the group of burdened and unaffected caregivers, we used an unpaired *t* test with independent variables for the evaluated predictors of caregiver burden. To correct for multiple comparisons, the significance level has been adjusted to 0.05/n. Descriptive statistics and Pearson-regression analyses were carried out using SPSS 25.0 (IBM, Armonk, NY).

## Results

### Caregiver characteristics

93 caregivers completed all questionnaires, 36.6% of them were female. Mean age of caregivers was 61.6 years (± 13.5, min 27, max 86). Most caregivers had a middle (37.6%) or low (34.4%) level of education, and 80.7% of them were spouses of the related dystonia patient.

**Fig. 1:**
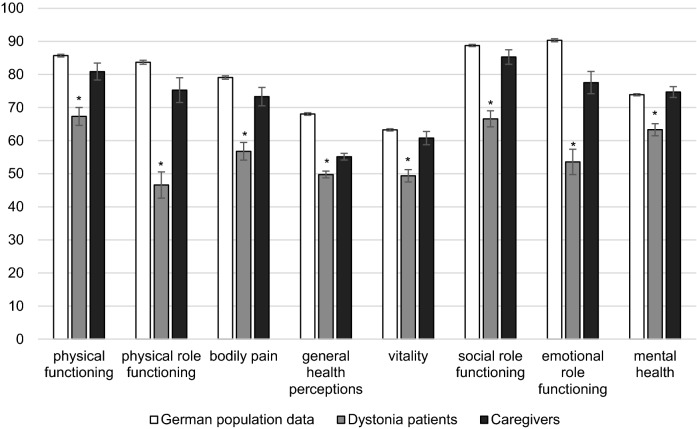
Health-related quality of life (HR-QoL) measured by Short Form 36 in patients with dystonia and their caregivers compared to the age-matched general German population. Bar graph comparing the HR-QoL of our study patients (light grey) and caregivers (black) with the general German population (data from 2003) (white). All SF-36 subscales are significantly reduced in the dystonia patients compared to the German age-matched population. Caregivers HR-QoL did not show significant differences in comparison to the German age-matched population. *p < 0.001

Caregivers spent an average of 2.2 h per day (± 5.3, min 0, max 24) with caregiving activities, e.g., support in mobility and self-care. On average the CBI score was 8.6 (± 9.6, min 0, max 48) suggesting no caregiver burden. Caregivers had a mean BDI score of 6.0 (± 5.9, min 0, max 26), indicating no depressive symptoms. The TAS-26 was on average 2.4 (± 0.5) not suggestive for alexithymia. In terms of anxiety, caregivers also showed an average degree of state and trait anxiety indicated by a mean STAI-S score of 38 (± 10.7, min 20, max 66), and an STAI-T score of 35.5 (± 9.9, min 21, max 63), respectively. All caregiver characteristics are summarized in Table 1. The caregivers’ mean SF-36 score was 72.5 points (± 17.9, min 24.2, max 95.3); for comparison with patients’ SF-36 data, see Fig. 1.

**Table 1:** Demographic and clinical data of caregivers

Item	Distribution	*N* = 93
Sex	*N* females	34 (36.6%)
	*N* males	59 (63.4%)
Age	Mean ± SD	61.6 ± 13.5
	Range	27–86
	*N* missing data	0
Educational level	High	22 (23.7%)
	Middle	35 (37.6%)
	Low	32 (34.4%)
	Missing data	4 (4.3%)
Relationship with patient	Spouses and life partner	75 (80.7%)
	Daughter/son	9 (9.7%)
	Sister/brother	2 (2.2%)
	Other related persons	5 (5.4%)
	Missing data	2 (2.2%)
Caregiving, h/day	Mean ± SD	2.2 ± 5.3
	Range	0–24
	Missing data	2 (2.2%)
CBI	Mean ± SD	8.6 ± 9.6
	Range	0–48
	Missing data	0
BDI	Mean ± SD	6 ± 5.9
	Range	0–26
	Missing data	11 (11.8%)
TAS-26	Mean ± SD	2.4 ± 0.5
	Range	1.3–3.7
	Missing data	4 (4.3%)
STAI-S	Mean ± SD	38 ± 10.7
	Range	20–66
	Missing data	3 (3.2%)
STAI-T	Mean ± SD	35.5 ± 9.9
	Range	21–63
	Missing data	2 (2.2%)
SF-36	Mean ± SD	72.5 ± 17.9
	Range	24.2–95.3
	Missing data	0

*CBI* Caregiver Burden Inventory, *BDI* Beck Depression Inventory, *TAS-26* Toronto-Alexithymia Scale, *STAI-S state anxiety, STAI-T trait anxiety* State–Trait–Anxiety Inventory, *SD* standard deviation, *SF-36* WHO Short Form 36 Health Survey

Overall, our results suggest only a low amount of caregiver burden. Therefore, we wanted to compare the caregivers who reported relevant burden (*N* = 9, CBI score ≥ 20) and the caregivers without (*N* = 84, CBI score < 20) in a subgroup analysis to identify additional potential risk factors for higher caregiver burden. In comparison with the unburdened caregivers, the group of burdened caregivers showed significantly higher BDI scores and lower HR-QoL, similar to their related patients (Table 2).

**Table 2:** Comparison of non-burdened caregivers’ characteristics with burdened caregivers’ characteristics

	Item	Non-burdened (CBI < 20)	Burdened (CBI ≥ 20)	Unpaired *t* test
		*N* = 84	*N* = 9	
Caregivers’ properties	Age	61.1 ± 13.5	66.7 ± 12.4	0.244
	CG h/d	1.9 ± 5.2	4.6 ± 6.0	0.162
	SF-36	74.2 ± 17.1	56.3 ± 16.0	0.004**
	BDI	5.3 ± 5.6	12.2 ± 5.1	0.001**
Dystonia patients’ properties	Age	63.9 ± 10.6	65 ± 16.0	0.853
	BFMDRS-I	11.3 ± 11.2	21.8 ± 22.5	0.234
	BFMDRS-II	2.2 ± 2.9	7.0 ± 9.8	0.205
	SF-36	57.7 ± 20.7	37.7 ± 10.6	0.001**
	BDI	10.2 ± 8.4	17.4 ± 8.9	0.075
	MoCA	25.8 ± 2.7	21.9 ± 5.1	0.124

This table displays the difference between non-burdened caregivers (CBI < 20) and burdened caregivers (CBI** ≥ **20). Non-burdened caregivers’ SF scores are significantly higher than burdened caregivers’ scores, also the SF-36 scores of the corresponding patients are significantly higher, BDI scores of the non-burdened caregivers are significantly lower than BDI scores of the burdened caregivers

Table 3 Unpaired *t* test (*p < 0.05, **p < 0.005) between caregivers with and without burden. Data of corresponding dystonia patients are shown above

*CBI* Caregiver Burden Inventory, *CG h/d* caregiving hours/day, *SF-36* WHO Short Form 36 Health Survey, *BDI* Beck Depression Inventory, *BFMDRS-I motor score, BFMDRS-II disability score* Burke–Fahn–Marsden Dystonia Rating Scale, *MoCA* Montreal Cognitive Assessment

We performed a univariate regression model for the association of the CBI score with sum scores of investigated predictors. The univariate regression with adjusted significance level of *p* < 0.004 showed significant correlations for the CBI score with the caregiving hours per day (*b* = 0.329, *r* = 0.409, *p* = 0.001), the patients’ average SF-36 scores (*b* = 0.392, *r* = − 0.379, *p* < 0.001), and the patients’ STAI-T (*b* = 0.333, *r* = 0.323, *p* = 0.001). Furthermore, the univariate regression showed significant correlations for the CBI score with the caregivers’ average SF-36 scores (*b* = 0.217, *r* = − 0.214, *p* = 0.038), the caregivers’ STAI-S (*b* = 0.225, *r* = 0.220, *p* = 0.033), the patients’ STAI-S (*b* = 0.295, *r* = 0.288, *p* = 0.005), the caregivers’ STAI-T (*b* = 0.240, *r *= 0.243, *p* = 0.022), the BFMDRS-I (*b* = 0.216, *r* = 0.215, *p* = 0.049), the BFMDRS-II (*b* = 0.280, *r* = 0.284, *p* = 0.010), and the patients’ TAS-26 score (*b* = 0.218, *r* = 0.211, *p* = 0.040). A back-to-back selection at a significance level of 5% was carried out for a multivariate regression model. After 8 runs, the caregiving hours per day (*b* = 0.313, *r* = 0.395, *p* = 0.03) and the patients’ STAI-T (*b* = 0.378, *r* = 0.366, *p* < 0.001) remained significantly correlated with the CBI.

### Patient characteristics

We screened 175 dystonia patients during data collection. 114 patients completed all questionnaires, 72.8% of them were females. On average, the included 114 patients were 64.0 years old (± 11.1 standard deviation, min 30, max 85). Mean time under diagnosis was 14 years (± 9.4, min 0, max 48). Patients’ average BFMDRS-I score was 11.7 (± 13) and the BFMDRS-II score was 2.4 (± 4). Patients suffering from a cervical dystonia had an average TWSTRS score of 27.4 (± 13.9).

The majority of the participants had a middle (43.0%) or low (42.1%) level of education. The cognitive function of the patients turned out to be normal with a mean MoCA score of 25.5 (± 3.1, min 12, max 30). A severe cognitive impairment, corresponding to an MoCA score below 21 points, was only found in 7.9% of all patients. Our patients had a mean BDI score of 10.7 (± 8.7, min 0, max 45) suggesting no depressive syndrome. However, 4.4% of our patients suffered from marked symptoms of depression, 7.9% from moderate, and 14.0% from mild depressive symptoms. Similarly, there was no sign of alexithymia in our patient sample. The TAS-26 was on average 2.5 (± 0.5, min 1.22, max 3.81) with no major difference to the standard cutoff for alexithymia of 2.4 (± 0.5) and below. In terms of anxiety, our patients showed an average degree of state and trait anxiety indicated by a mean STAI-S score of 40.5 (± 12.1, min 22, max 69), and an STAI-T score of 40.8 (± 11, min 21, max 69). All patient characteristics are summarized in Table 3.

**Table 3:** Demographic and clinical data of dystonia patients

Item	Distribution	*N* = 114
Sex	*N* females	83 (72.8%)
	*N* males	31 (27.2%)
Age	Mean ± SD	64 ± 11.1
	Range	30–85
	*N* missing data	2 (1.8%)
Educational level	High	14 (12.3%)
	Middle	49 (43%)
	Low	48 (42.1%)
	Missing data	3 (2.6%)
Years since diagnosis	Mean ± SD	14 ± 9.4
	Range	0–48
	Missing data	8 (7%)
BFMDRS-I	Mean ± SD	11.7 ± 13
	Range	0.5–72
	Missing data	11 (9.7%)
BFMDRS-II	Mean ± SD	2.4 ± 4
	Range	0–29
	Missing data	11 (9.7%)
TWSTRS (only cervical dystonia patients)	Mean ± SD	27.4 ± 13.9
	Range	0–63.5
	Missing data	10 (15.2%)
MoCA total score	Mean ± SD	25.5 ± 3.1
	Range	12–30
	Missing data	12 (10.5%)
BDI	Mean ± SD	10.7 ± 8.7
	Range	0–45
	Missing data	1 (0.9%)
TAS-26	Mean ± SD	2.5 ± 0.5
	Range	1.2 3.8
	Missing data	3 (2.6%)
STAI-S	Mean ± SD	40.5 ± 12.1
	Range	22–69
	Missing data	2 (1.8%)
STAI-T	Mean ± SD	40.8 ± 11
	Range	21–69
	Missing data	2 (1.8%)
SF-36	Mean ± SD	56.3 ± 20.8
	Range	10.4–94.9
	Missing data	0 (0%)

*BFMDRS-I motor score, BFMDRS-II disability score* Burke–Fahn–Marsden Dystonia Rating Scale, *TWSTRS* Toronto Western Spasmodic Torticollis Rating Scale, *MoCA* Montreal Cognitive Assessment, *BDI* Beck Depression Inventory, *TAS-26* Toronto-Alexithymia Scale, State–Trait–Anxiety Inventory, standard deviation

We investigated 89 patients with focal, 16 with segmental, and 9 with generalized dystonia. Table 4 displays all dystonia subgroups included in our study (multiple subtypes were possible, e.g., focal and blepharospasm). Across all subgroups, our patients had an average SF-36 score of 56.3 (± 28.3, min 0, max 100). The HR-QoL is decreased compared to a general age-matched German population. Figure 1 displays the patients’ SF-36 sub scores, the age-matched German population data from 2003, and, additionally, caregivers’ HR-QoL (Kurth and Ellert 2002). It is notable that, on average, patients with focal dystonia had higher SF-36 scores than patients with segmental or generalized dystonia (Table 4).

**Table 4:** Clinical data of dystonia subtypes in our cohort (*N* = 114 patients, multiple subtypes possible, e.g., focal and blepharospasm)

Dystonia	*N*	Item	Mean ± SD	Range
Focal	89	BFMDRS-I	8.2 ± 8.3	0.5–58
		BFMDRS-II	1.4 ± 2.1	0–11
		SF-36	58.8 ± 20.8	19.6–94.9
Segmental	16	BFMDRS-I	12.2 ± 5	4–20
		BFMDRS-II	2.4 ± 1.9	0–6
		SF-36	50.6 ± 18.5	22.5–81.7
Generalized	9	BFMDRS-I	41.6 ± 17.5	16.5–72
		BFMDRS-II	10.8 ± 7.4	3–29
		SF-36	43 ± 17.8	10.4–69.9
Blepharospasm	16	BFMDRS-I	5.8 ± 4.3	1–20
		BFMDRS-II	0.6 ± 1.4	0–6
		SF-36	56.4 ± 21.1	29.6–89.9
Cervical dystonia	66	BFMDRS-I	8.9 ± 9.3	0.5–58
		BFMDRS-II	1.5 ± 2.2	0–11
		SF-36	58.5 ± 20.2	19.6–94.9
		TWSTRS	26.2 ± 12.7	3–57
		CDQ-24	33.4 ± 19.1	0–86.2

*BFMDRS-I motor score, BFMDRS-II disability score* Burke–Fahn–Marsden Dystonia Rating Scale, *MoCA* Montreal Cognitive Assessment, *BDI* Beck Depression Inventory, *TWSTRS* Toronto Western Spasmodic Torticollis Rating Scale, *CDQ-24* Craniocervical Dystonia Questionnaire, *SD* standard deviation

We performed a univariate regression model for the association of average SF-36 scores with sum scores of investigated predictors. Our univariate regression analysis with adjusted significance level of *p* < 0.006 showed significant negative correlations for the patients’ BDI (*b* = 0.352, *r* = − 0.531, *p* < 0.001), STAI-S (*b* = 0.407, *r* = − 0.414, *p* < 0.001), and STAI-T score (*b* = 0.358, *r* = − 0.359, *p* < 0.001). Furthermore, the univariate regression showed significant negative correlations for the patients’ average SF-36 score, the disability scale BFMDRS-II (*b* = 0.198, *r* = − 0.211, *p* = 0.047), and TAS-26 score (*b* = 0.211, *r *= − 0.210, *p* = 0.028). To test against co-correlations and confounding of patients’ variables, the BFMDRS-II, BDI, TAS-26, STAI-S, and STAI-T, which were significant in the univariate model, were examined in a multivariate model. A back-to-back selection at a significance level of 5% was carried out. After six runs, STAI-S (*b* = 0.296, *r* = − 0.306, *p* = 0.006) and BDI scores (*b *= 0.220, *r* = − 0.333, *p* = 0.041) were significantly correlated with patients’ HR-QoL.

## Discussion

In the present study, we examined caregiver burden in a group of dystonia patients and their caregivers, together with the HR-QoL of patients and their caregivers. Patients’ trait anxiety (as measured by the STAI) as well as the number of caregiving hours per day emerged as predictors of unique variance in the burden experienced by caregivers of patients with dystonia. In addition, we found that, even under treatment with BT, dystonia patients have a decreased HR-QoL in all SF-36 sub scores (Fig. 1) compared to their caregivers and the general German population (Kurth and Ellert 2002). Our statistical analysis shows a significant negative correlation between lower SF-36 and higher STAI-S and BDI scores of the patients. Hence, depression and state anxiety seem to account for HR-QoL in dystonia, similar data could already be shown in Parkinson’s disease (Aarsland et al. 1999, 2007; Carod-Artal et al. 2013; Martínez-Martín et al. 2007).

### Caregiver burden

In Parkinson’s disease, caring for a family member has been recognized to negatively impact the caregivers’ life in physical, emotional, and psychosocial aspects, associated with an increased risk of psychiatric morbidity and persistent distress (Greenwell et al. 2014). Non-motor symptoms of the patient consistently make a greater contribution to caregiver burden than motor symptoms. In particular, patients’ anxiety (Carod-Artal et al. 2013; Leiknes et al. 2010), depression (Aarsland et al. 1999; Martínez-Martín et al. 2007), and cognitive impairment (Coelho et al. 2015) are an important predictor of caregiver burden. The identification and timely treatment of caregiver burden also have a substantial economic benefit for society in addition to the health-related benefits to the patient (Martinez-Martin et al. 2019a).

So far, to our knowledge, no data on caregiver burden have been available for dystonia. In our large sample, we found only a low amount of caregiver burden. Factors explaining this could be the preserved patients’ autonomy due to the treatment with BT and the low amount of daily caregiving. The latter explanation is supported by the results of our multivariate regression analysis, which demonstrated that the number of caregiving hours is positively linked to reported caregiver burden. In addition, this analysis identified depression and cognitive impairment as unique predictors of caregiver burden, suggesting that, for the few caregivers with relevant burden (CBI > 20), the related patients suffered from even less HR-QoL, more depression and slightly more cognitive impairment. The limited variance in detected caregiver burden might underestimated the role of possible variance-explaining predictors. There was no particular type of dystonia associated with higher caregiver burden, but the corresponding patients had more severe dystonic symptoms as assessed by the BFMDRS-I and BFMDRS-II. Further research should focus on major groups of patients with generalized dystonia or more severe dystonic symptoms.

### HR-QoL in dystonia patients

In general, dystonia is associated with a reduction of HR-QoL (Mordin et al. 2014). Patients suffering from dystonia reported more problems with daily activities, e.g., mobility and self-care (Gudex et al. 1998). In addition, only a minority of the patients reported complete satisfaction with their treatment at the end of a botulinum toxin (BT) therapy cycle as recently shown in large study groups (Colosimo et al. 2019; Misra et al. 2018). This is in line with our observation on decreased HR-QoL, as we detected a persistent reduction in patients’ SF-36 scores, even under chronic treatment with BT, compared to general German population data. While we used age-matched control data, our study group had a slightly higher female proportion of 72.8%. Since women tend to achieve lower SF-36 scores (Ellert and Kurth 2013), the difference between our data and the control cohort may actually be smaller. However, if the data for an all-female control are taken as a basis, the effect remains clearly significant (data not shown).

Several previous studies also found a high prevalence of depression and anxiety in patients with dystonia (Gündel et al. 2003; Moraru et al. 2002; Wenzel et al. 1998), which emphasizes these symptoms as an important factor in the disease process. Our statistical analysis also indicated that the reduced HR-QoL is mainly associated with symptoms beyond motor impairment, such as depression and anxiety. This is in line with Ben-Shlomo et al. (2002), Conte et al. (2015) and Page et al. (Page et al. 2007), who have already suggested that these neuropsychological symptoms are the main predictors for HR-QoL in dystonia. Similar results were found for patients with Parkinson’s disease (Aarsland et al. 2009; Klietz et al. 2018).

Remarkably, the severity of dystonia, as measured by the BFMDRS-I or TWSTRS, showed no statistically significant correlation with HR-QoL, although all patients were examined in the wearing-off of the BT treatment with probably the greatest extent of motor symptoms. In addition, patients’ treatment satisfaction seemed to be stable, even if clinical features were improved as measured by TWSTRS and Tsui scale (Colosimo et al. 2019). This matches prior results, suggesting that severity of dystonia is a weaker predictor for HR-QoL in dystonia than depression and anxiety (Ben-Shlomo et al. 2002; Page et al. 2007). Similar results have been obtained by Smit et al. (2016). Czekóová et al. (2017) found a reduced emotional awareness or alexithymia in patients with cervical dystonia, suggesting that this could be another predictor for reduced HR-QoL. However, we did not find a relevant proportion of alexithymia as measured by the TAS-26 in our sample. When treated with BT, motor symptoms may be negligible as main predictor of HR-QoL in most dystonia patients. Since these non-motor symptoms are not fully addressed by BT, these symptoms seemed to be predominant in our patients undergoing chronic BT treatment and were associated with poorer HR-QoL. It, therefore, seems useful for patients suffering from dystonia, in addition to an assessment of motor symptoms, to carry out an assessment of the coexisting non-motor symptoms (depression, anxiety, cognitive impairment). A quick and clinically useful self-assessment, similar to the Movement Disorder Society Non-motor Scale (MDS-NMS) for Parkinson’s disease (Martinez-Martin et al. 2019b), was recently published for dystonia patients (Klingelhoefer et al. 2019). Our results provide further evidence for the profound impact of dystonia on patients’ HR-QoL. The mismatch between objective BT-induced improvement of dystonia and HR-QoL, which still seems to be affected, as determined by the SF-36, illustrates the need for a specific therapy of these non-motor symptoms. In the long run, it might prove beneficial to support patients by pharmacological and/or psychological treatment, so that they will be able to cope with their neuropsychological symptoms and restriction of activities of daily living.

Further large-scale studies with de novo patients are needed to examine neuropsychological symptoms, pain, and sleep disorders, and their impact on HR-QoL in dystonia.

### Limitation

Despite the fact that we examined a large cohort of patients with dystonia and their caregivers, a limiting factor remains the different group sizes of the dystonia subtypes. In addition, our study cohort contains hardly any de novo patients, so there is no direct comparison to the reduced HR-QoL of untreated dystonia patients and their caregivers.

Even though we could show substantial evidence of a decreased HR-QoL in our patients, only low caregiver burden could be detected. The CBI as a measuring instrument for caregiver burden is not specific to dystonia, but nevertheless, the gold standard for evaluating caregiver burden internationally.

Another limitation arises from the small number of caregivers for generalized dystonia patients who reported a higher caregiver burden. In future studies, a multicentric approach should enable to investigate more patients with generalized dystonia and their caregivers.

### Conclusion

In our patients with dystonia, the related caregiver burden appeared to be low and only mildly related to HR-QoL of the caregiver. For a small subgroup of patients with marked dystonic symptoms and pronounced HR-QoL deficits, burden of the corresponding caregiver seemed to be an issue. Therefore, we propose to perform a corresponding assessment when caregiver burden is suspected. To implement an individual intervention, an improved caregiver education about the patients’ disease, a reduction of the daily caregiving hours, and a better social support can be established. To our knowledge, this is the first caregiver burden study in a larger group of patients with dystonia. Further studies are needed to describe caregiver burden in the subgroup of patients suffering from severe dystonia.

Notwithstanding the fact that all dystonia patients received optimized treatment with BT, their HR-QoL was still reduced. Despite a relief of their symptoms, they still suffered from dystonic symptoms and a chronic disease. This might partly explain the reduction in HR-QoL and more severe depressive symptoms in the patient cohort. Overall, our data indicate that effective treatment of dystonia patients has to include screening and management of neuropsychiatric symptoms which should be addressed in future multicentric studies.

## Electronic supplementary material

Below is the link to the electronic supplementary material.

Supplementary material 1 (DOCX 13 kb)
