# Supplementary material for: Caregiver burden and health-related quality of life in idiopathic dystonia patients under botulinum toxin treatment: a cross-sectional study
Source: J Neural Transm (Vienna). 2019 Dec 4;127(1):61–70. doi: 10.1007/s00702-019-02109-6 (PMC6942568; doi:10.1007/s00702-019-02109-6)
Supplement: Supplementary file 1 — Supplementary material 1 (DOCX 13 kb) [file 702_2019_2109_MOESM1_ESM.docx]

**Supplements**

Supplement 1: Predicting factors for patients’ average SF-36 scores.

| **predicting factors** | **r** | **b** | **p** |
| --- | --- | --- | --- |
| patients’ age | -0.074 | -0.074 | 0.441 |
| patients’ level of education | 0.088 | 0.087 | 0.910 |
| BFMDRS-I scores | -0.184 | -0.174 | 0.081 |
| BFMDRS-II scores | -0.211 | -0.198 | 0.047 * |
| patients’ MoCA total scores | 0.060 | 0.042 | 0.682 |
| patients’ BDI scores | -0.531 | -0.352 | <0.001 ** |
| patients’ TAS-26 scores | -0.210 | -0.211 | 0.028 * |
| patients’ STAI-S scores | -0.414 | -0.407 | <0.001 ** |
| patients’ STAI-T scores | -0.359 | -0.358 | <0.001 ** |

Abbreviations: Burke-Fahn-Marsden Dystonia Rating Scale (BFMDRS-I motor score, BFMDRS-II disability score), Montreal Cognitive Assessment (MoCA), Beck Depression Inventory (BDI), Toronto-Alexithymia-Scale (TAS-26), State-Trait-Anxiety Inventory (STAI-S state anxiety, STAI-T trait anxiety). * indicates p<0.05, ** indicates p<0.006 (To correct for multiple comparisons the significance level has been adjusted to p<0.006).

Supplement 2: Predicting factors for caregivers’ CBI scores.

| **predicting factors** | **r** | **b** | **p** |
| --- | --- | --- | --- |
| age difference | -0.057 | -0.052 | 0.687 |
| caregiving hours per day | 0.409 | 0.329 | 0.001 ** |
| caregivers’ level of education | -0.038 | -0.037 | 0.728 |
| caregivers’ BDI scores | 0.195 | 0.197 | 0.076 |
| caregivers’ average SF-36 scores | -0.214 | -0.217 | 0.038 * |
| caregivers’ TAS-26 scores | 0.178 | 0.182 | 0.088 |
| caregivers’ STAI-S scores | 0.220 | 0.225 | 0.033 * |
| caregivers’ STAI-T scores | 0.243 | 0.240 | 0.022 * |
| BFMDRS-I scores | 0.215 | 0.216 | 0.049 * |
| BFMDRS-II scores | 0.284 | 0.280 | 0.010 * |
| patients’ average SF-36 scores | -0.379 | -0.392 | <0.001 ** |
| patients’ STAI-S scores | 0.288 | 0.295 | 0.005 * |
| patients’ STAI-T scores | 0.323 | 0.333 | 0.001 ** |
| patients’ TAS-26 scores | 0.211 | 0.218 | 0.040 * |

Abbreviations: Beck Depression Inventory (BDI), WHO Short Form 36 (SF-36) Health Survey, Toronto-Alexithymia-Scale (TAS-26), State-Trait-Anxiety Inventory (STAI-S state anxiety, STAI-T trait anxiety), Burke-Fahn-Marsden Dystonia Rating Scale (BFMDRS-I motor score, BFMDRS-II disability score). * indicates p<0.05, ** indicates p<0.004 (To correct for multiple comparisons the significance level has been adjusted to p<0.004).
